# 108 Effects of Prior Hyperthyroidism Diagnosis on Burn Patient Outcomes

**DOI:** 10.1093/jbcr/irad045.081

**Published:** 2023-05-15

**Authors:** Chris Soudah, Kathleen Karam, Dalton Amador, Isabel Obias, Amy Arceneaux, Steven Wolf, Juquan Song, Amina El Ayadi, Georgiy Golovko

**Affiliations:** The University of Texas Medical Branch, Galveston, Texas; The University of Texas Medical Branch, Galveston, Texas; The University of Texas Medical Branch, Galveston, Texas; The University of Texas Medical Branch, Galveston, Texas; The University of Texas Medical Branch, Galveston, Texas; The University of Texas Medical Branch, Galveston, Texas; The University of Texas Medical Branch, Galveston, Texas; The University of Texas Medical Branch, Galveston, Texas; The University of Texas Medical Branch, Galveston, Texas; The University of Texas Medical Branch, Galveston, Texas; The University of Texas Medical Branch, Galveston, Texas; The University of Texas Medical Branch, Galveston, Texas; The University of Texas Medical Branch, Galveston, Texas; The University of Texas Medical Branch, Galveston, Texas; The University of Texas Medical Branch, Galveston, Texas; The University of Texas Medical Branch, Galveston, Texas; The University of Texas Medical Branch, Galveston, Texas; The University of Texas Medical Branch, Galveston, Texas; The University of Texas Medical Branch, Galveston, Texas; The University of Texas Medical Branch, Galveston, Texas; The University of Texas Medical Branch, Galveston, Texas; The University of Texas Medical Branch, Galveston, Texas; The University of Texas Medical Branch, Galveston, Texas; The University of Texas Medical Branch, Galveston, Texas; The University of Texas Medical Branch, Galveston, Texas; The University of Texas Medical Branch, Galveston, Texas; The University of Texas Medical Branch, Galveston, Texas; The University of Texas Medical Branch, Galveston, Texas; The University of Texas Medical Branch, Galveston, Texas; The University of Texas Medical Branch, Galveston, Texas; The University of Texas Medical Branch, Galveston, Texas; The University of Texas Medical Branch, Galveston, Texas; The University of Texas Medical Branch, Galveston, Texas; The University of Texas Medical Branch, Galveston, Texas; The University of Texas Medical Branch, Galveston, Texas; The University of Texas Medical Branch, Galveston, Texas; The University of Texas Medical Branch, Galveston, Texas; The University of Texas Medical Branch, Galveston, Texas; The University of Texas Medical Branch, Galveston, Texas; The University of Texas Medical Branch, Galveston, Texas; The University of Texas Medical Branch, Galveston, Texas; The University of Texas Medical Branch, Galveston, Texas; The University of Texas Medical Branch, Galveston, Texas; The University of Texas Medical Branch, Galveston, Texas; The University of Texas Medical Branch, Galveston, Texas; The University of Texas Medical Branch, Galveston, Texas; The University of Texas Medical Branch, Galveston, Texas; The University of Texas Medical Branch, Galveston, Texas; The University of Texas Medical Branch, Galveston, Texas; The University of Texas Medical Branch, Galveston, Texas; The University of Texas Medical Branch, Galveston, Texas; The University of Texas Medical Branch, Galveston, Texas; The University of Texas Medical Branch, Galveston, Texas; The University of Texas Medical Branch, Galveston, Texas; The University of Texas Medical Branch, Galveston, Texas; The University of Texas Medical Branch, Galveston, Texas; The University of Texas Medical Branch, Galveston, Texas; The University of Texas Medical Branch, Galveston, Texas; The University of Texas Medical Branch, Galveston, Texas; The University of Texas Medical Branch, Galveston, Texas; The University of Texas Medical Branch, Galveston, Texas; The University of Texas Medical Branch, Galveston, Texas; The University of Texas Medical Branch, Galveston, Texas; The University of Texas Medical Branch, Galveston, Texas; The University of Texas Medical Branch, Galveston, Texas; The University of Texas Medical Branch, Galveston, Texas; The University of Texas Medical Branch, Galveston, Texas; The University of Texas Medical Branch, Galveston, Texas; The University of Texas Medical Branch, Galveston, Texas; The University of Texas Medical Branch, Galveston, Texas; The University of Texas Medical Branch, Galveston, Texas; The University of Texas Medical Branch, Galveston, Texas; The University of Texas Medical Branch, Galveston, Texas; The University of Texas Medical Branch, Galveston, Texas; The University of Texas Medical Branch, Galveston, Texas; The University of Texas Medical Branch, Galveston, Texas; The University of Texas Medical Branch, Galveston, Texas; The University of Texas Medical Branch, Galveston, Texas; The University of Texas Medical Branch, Galveston, Texas; The University of Texas Medical Branch, Galveston, Texas; The University of Texas Medical Branch, Galveston, Texas

## Abstract

**Introduction:**

The metabolic response to severe burns is described by an initial hypometabolic ‘ebb’ phase and followed by a hypermetabolic ‘flow’ phase. The hypermetabolic phase is mediated by hormonal and cellular dysregulation with elevated catecholamine levels. The role of thyroid hormone, however, is not described well. This study investigates relative risks of various outcomes in those with a previous diagnosis of hyperthyroidism following severe burn compared to those without.

**Methods:**

This study obtained data from the TriNetX Research Network, a national database of de-identified electronic medical data from a secure network. The population was stratified to compare patients with and without a prior diagnosis of hyperthyroidism who sustained burns. Outcomes were measured within three years of injury in order to assess long-term effects occurring alongside the hypermetabolic phase. Outcomes observed for both groups included systemic inflammation and infection, sepsis, mortality, and infections of the skin and subcutaneous tissue.

**Results:**

This study identified 5,250 patients with a prior diagnosis of hyperthyroidism who sustained burn injuries and 648,336 patients without hyperthyroidism prior to matching for demographics and diagnoses. Propensity score matching then yielded 5,077 patients with and without a prior diagnosis of hyperthyroidism. Both cohorts were balanced using propensity score matching for age, sex, race, ethnicity, and TBSA burned.

After assessing the outcomes for a three-year span since burn injury, patients with a prior diagnosis of hyperthyroidism had a higher risk of systemic inflammation and infection (risk ratio [RR], 1.599, p < 0.0003), sepsis ([RR], 1.572, p < 0.0001), mortality ([RR], 1.488, p < 0.0001), and developing infections of the skin and subcutaneous tissue ([RR], 1.332, p < 0.0001) compared to patients without prior diagnosis history.

**Conclusions:**

Burned patients with a prior history of hyperthyroidism have increased risk of systemic inflammation and infection, sepsis, mortality, and infections of the skin and subcutaneous tissue. Thus, history of this condition or concomitant diagnosis with the injury is warranted.

**Applicability of Research to Practice:**

This study encourages further insight into the relationship between the metabolic effects of burn injuries and concurrent metabolic conditions to modify patient treatment plans and customize practices.

